# Forkhead box L2 is a target of miR‐133b and plays an important role in the pathogenesis of non‐small cell lung cancer

**DOI:** 10.1002/cam4.5746

**Published:** 2023-02-27

**Authors:** Juan Li, Lirong Gao, Anqi Wang, Huiwen Qian, Jianjie Zhu, Shundong Ji, Jun Chen, Zeyi Liu, Cheng Ji

**Affiliations:** ^1^ Department of Pulmonary and Critical Care Medicine The First Affiliated Hospital of Soochow University Suzhou China; ^2^ Suzhou Key Laboratory for Respiratory Diseases Suzhou China; ^3^ Department of Respiratory and Critical Care Medicine Affiliated Hospital of Nantong University Nantong China; ^4^ Institute of Respiratory Diseases Soochow University Suzhou China; ^5^ Jiangsu Institute of Hematology, MOH Key Laboratory of Thrombosis and Hemostasis, Collaborative Innovation Center of Hematology The First Affiliated Hospital of Soochow University Suzhou China; ^6^ Department of Thoracic Surgery The First Affiliated Hospital of Soochow University Suzhou China

**Keywords:** carcinoma, non‐small‐cell lung cancer (NSCLC), epithelial–mesenchymal transition (EMT), forkhead box protein L2 (FOXL2), miR‐133b, TGF‐β/Smad signaling

## Abstract

**Background:**

Forkhead box L2 (FOXL2) has been recognized as a transcription factor in the progression of many malignancies, but its role in non‐small cell lung cancer (NSCLC) remains unclear. This research clarified on the role of FOXL2 and the specific molecular mechanism in NSCLC.

**Methods:**

RNA and protein levels were detected by quantitative real‐time polymerase chain reaction (qRT–PCR) and western blotting assays. Cell proliferation was examined by cell counting kit‐8 (CCK‐8) and clonogenic assays. Transwell and wound healing assays were used to detect cell invasion and migration. Cell cycle alterations were assessed by flow cytometry. The relationship between FOXL2 and miR‐133b was verified by dual‐luciferase reporter assays. In vivo metastasis was monitored in the tail vein‐injected mice.

**Results:**

FOXL2 was upregulated in NSCLC cells and tissues. Downregulation of FOXL2 restrained cell proliferation, migration, and invasion and arrested the cell cycle of NSCLC cells. Moreover, FOXL2 promoted the epithelial–mesenchymal transition (EMT) process of NSCLC cells by inducing the transforming growth factor‐β (TGF‐β)/Smad signaling pathway. miR‐133b directly targeted the 3′‐UTR of FOXL2 and negatively regulated FOXL2 expression. Knockdown of FOXL2 blocked metastasis in vivo.

**Conclusions:**

miR‐133b downregulates FOXL2 by targeting the 3′‐UTR of FOXL2, thereby inhibiting cell proliferation, EMT and metastasis induced by the TGF‐β/Smad signaling pathway in NSCLC. FOXL2 may be a potential molecular target for treating NSCLC.

## Graphical abstract

FOXL2 promotes cell proliferation and metastasis in NSCLCmiR‐133b downregulates FOXL2 by targeting the 3’‐UTR of FOXL2.

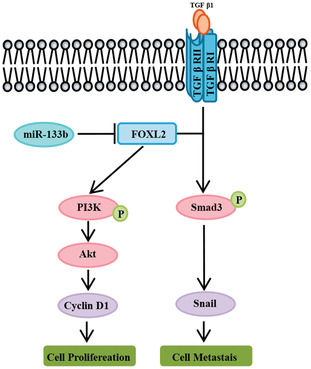

## 1 INTRODUCTION

Lung cancer remains one of the most pervasive tumors and accounts for approximately one in five (18.4%) cancer‐related deaths annually worldwide.^1^, ^2^, ^3^, ^4^, ^5^ Non‐small cell lung cancer (NSCLC) accounts for approximately 85% of lung cancer cases among common subtypes.^4^ Due to the insidious early symptoms of NSCLC, approximately 70% of cases already have distant or local metastasis by the time of diagnosis.^6^ Advanced NSCLC patients have obtained great benefits from the progressive treatments available, including epidermal growth factor receptor tyrosine kinase inhibitors (EGFR‐TKIs),^7^ antiangiogenic agents,^8^ and immune checkpoint inhibitors.^9^ However, the prognosis for NSCLC remains unsatisfactory.^6^ Only 70% of patients with EGFR mutations are sensitive to EGFR‐TKI therapy. These patients inevitably develop drug resistance after receiving EGFR‐TKI treatment for a period of time.^10^, ^11^ Only 20% of NSCLC patients with EGFR wild‐type or ALK fusion‐negative NSCLC responded to immunotherapy.^9^, ^12^, ^13^ Therefore, it is urgent to explore the etiopathogenesis of NSCLC and seek promising molecular targets for treating NSCLC.

The forkhead box (FOX) family is a large group of transcription factors that has been categorized into 19 subgroups, from FOXA to FOXS.^14^, ^15^, ^16^ Previous publications have found that FOX proteins are associated with numerous cancers, and their role could be either tumor‐suppressive or oncogenic based on the family member and cell category.^15^, ^17^ Forkhead box L2 (FOXL2) is a single‐exon gene that encodes a 376 amino acid transcription factor with a forkhead domain.^18^, ^19^ FOXL2 plays an important role in ovarian differentiation and development, and eyelid advance.^20^, ^21^, ^22^, ^23^ Its dysregulation could cause blepharophimosis‐ptosis‐epicanthus inversus syndrome, premature ovarian failure, or ovarian granulosa cell tumors (GCT).^24^, ^25^, ^26^ Emerging evidence suggests that FOXL2 is closely associated with tumors. FOXL2 dysfunction affects cell cycle progression, and apoptosis in ovarian GCT.^27^ Furthermore, FOXL2 expression was discovered to be abnormally high in gastric carcinoma and to boost cell proliferation as well as epithelial–mesenchymal transition (EMT)‐induced metastasis in chemoresistant gastric cancer.^28^, ^29^ FOXL2 expression is significantly higher in stage I NSCLC than in noncancerous lung diseases.^30^ Moreover, as one of the genes for hotspot mutation variants, FOXL2 was examined for molecular analysis in advanced NSCLC to seek novel targeted treatment options.^31^ However, the role of FOXL2 in NSCLC remains largely unknown. It would be interesting to determine the function of FOXL2 and the specific molecular mechanism in NSCLC.

EMT refers to the process in which cells gradually lose the adhesion properties of epithelial cells, separate from surrounding cells, and transform into mesenchymal cells.^32^ EMT transcription factors (Snail, Twist, ZEB), mesenchymal markers (N‐cadherin, Vimentin), and proteolysis markers (MT‐MMPs, MMPs) are upregulated, while the expression of epithelial markers (E‐cadherin, Epcam) are downregulated.^33^, ^34^ EMT programs have been divided into three types according to the biological context in which they occur.^35^ Type I EMT is related to embryonic development and organ formation, type II EMT is essential for wound healing and fibrosis, and type III EMT promotes tumor progression and metastasis.^36^, ^37^, ^38^ EMT promotes cell migration, invasion, and metastasis by enhancing cell motility and is linked to poor prognosis in several tumors.^39^ EMT can be induced by a variety of signaling pathways, the most classic of which is the transforming growth factor‐β (TGF‐β)/Smad signaling pathway. In the classic TGF‐β/Smad signaling pathway, TGF‐β can bind to TGF‐β receptor II on the cell membrane, which in turn recruits, phosphorylates and activates TGF‐β receptor I. Activated TGF‐β RI phosphorylates R‐Smads (Smad2 and Smad3), which can form the R‐Smad‐co‐Smad trimeric complex with co‐Smad (Smad4). Then, the complex migrates into the nucleus and acts as a transcription factor to regulate gene expression and induce the occurrence of EMT.^36^, ^40^ Some FOX proteins are involved in the EMT process in tumors. FOXK1 regulates cell proliferation and EMT‐induced metastasis by negatively regulating miR‐646 in gastric cancer.^41^ FOXM1D promotes EMT and metastasis of colorectal cancer by directly binding to and activating Rho‐related protein kinases.^42^ A recent study revealed that FOXL2 could promote EMT and metastasis of chem‐resistant gastric cancer through the HMGA2‐FOXL2‐ITGA2 signaling axis.^28^ Therefore, we wondered whether FOXL2 was involved in EMT and the TGF‐β/Smad signaling pathway in NSCLC.

MicroRNAs (miRNAs) are a class of conserved, short single‐stranded noncoding RNAs composed of 21–25 nucleotides that regulate gene expression after transcription.^43^, ^44^, ^45^ Mature miRNA chains can be integrated into RNA‐induced silencing complexes that specifically bind to the 3′‐untranslated region (3′‐UTR) of target gene mRNA to silence‐related target genes by degrading mRNA or preventing mRNA translation.^45^, ^46^ MiR‐133b is downregulated in many cancers; it inhibits cell proliferation and metastasis in breast, kidney, and prostate cancers, and is negatively correlated with prognosis.^47^, ^48^, ^49^ Our previous study showed that the miR‐133b levels in lung cancer tissues were much lower than those in noncancerous tissues according to the miRNA microarray in four paired NSCLC and normal tissues.^50^ Dai et al. reported that miR‐133b bound to the FOXL2 mRNA seed sequence in the 3′‐UTR to inhibit FOXL2 expression in human and mouse granulosa cells.^51^ Thus, it is highly likely that miR‐133b is an upstream regulator of the FOXL2 gene in NSCLC.

In the current study, we demonstrated that miR‐133b downregulated FOXL2 expression by targeting the 3′‐UTR of FOXL2 to inhibit cell proliferation, EMT, and metastasis induced by the TGF‐β/Smad signaling pathway in NSCLC. FOXL2 may be a potential molecular target for treating NSCLC.

## 2 MATERIALS AND METHODS

### 2.1 Tissue samples

We obtained 38 paired NSCLC and adjacent nonmalignant samples from the First Affiliated Hospital of Soochow University. All patients were diagnosed with NSCLC based on their histological and pathological characteristics according to the Revised International System for Staging Lung Cancer. No patients had received radiotherapy or chemotherapy before surgery. The collected tissue samples were frozen at −80°C for further study. This study was approved by the Ethics Committee of the First Affiliated Hospital of Soochow University. The corresponding ethical approval code is 2020‐375.

### 2.2 Cell culture

Human NSCLC cell lines (A549, H460, SPC‐A1, H1299, H1650, H1975, PC‐9) and human immortalized normal epithelial cells (BEAS‐2B) were purchased from the Cell Bank of the Chinese Academy of Sciences (Shanghai, China). Roswell Park Memorial Institute 1640 (RPMI 1640) or Dulbecco's modified Eagle medium (DMEM)/HIGH GLUCOSE medium (HyClone) containing 10% fetal bovine serum (FBS) (Gibco), 100 U/mL penicillin, and 100 μg/mL streptomycin were used to culture cells. All the cells were grown at 37°C in a humidified atmosphere with 5% CO_2_.

### 2.3 RNA interference

MiR‐133b mimics, miR‐133b inhibitors, and the related negative control (NC) were purchased from GenePharma. Two predesigned small interfering RNA (siRNA) sequences of FOXL2 were synthesized by GenePharma. The siRNA constructs were as follows: si‐FOXL2‐1: 5′‐GCUACCGCAGCCUCCCUCATT‐3′; si‐FOXL2‐2: 5′‐GCGUAGUGAACUCGUACAATT‐3′. We transfected siRNA into cells with Lipofectamine 2000 reagent (Invitrogen) according to the manufacturer's instructions.

### 2.4 Establishment of stable FOXL2‐silenced cell lines

To establish stable cell lines with silenced FOXL2 expression, one DNA fragment (FOXL2 shRNA, 5′‐GCUACCGCAGCCUCCCUCATT‐3′) was subcloned into the lentiviral vector piLenti‐siRNA‐GFP (GenePharma) containing endonuclease Bbsl and Swal. Then, the packaged lentiviruses were transduced into NSCLC cells. After 48 h, the infected cells were selected with 0.4 μg/mL puromycin (Sigma–Aldrich).

### 2.5 RNA extraction, cDNA synthesis, and quantitative real‐time polymerase chain reaction (qRT–PCR) analysis

Total RNA was extracted from cells by adding 1.0 mL of RNAiso Plus kit (Takara) according to the manufacturer's protocol. Reverse transcriptase M‐MLV (Takara) was used to synthesize cDNA. Primers for FOXL2, GAPDH, miR‐133b, and U6, which were used for reverse transcription and amplification, were purchased from Ribobio. 2×SYBR‐Green qPCR SuperMix (High ROX) (Bimake) and an ABI Step One Plus Real‐Time PCR system (Applied Biosystems) were used to conduct qRT–PCR according to the manufacturer's instructions. Ct values for FOXL2 were normalized to the internal control GAPDH. Ct values for miR‐133b were standardized to the internal control U6. The primers for FOXL2 and GAPDH were as follows: FOXL2, Forward: 5′‐CGCTGTCCGGCATCTACCAG‐3′ and Reverse: 5′‐CCGGGTCCAGCGTCCAGTAG‐3′; and GAPDH, Forward: 5′‐TGCACCACCAACTGCTTAGC‐3′ and Reverse: 5′‐GGCATGGACTGTGGTCATGG‐3′. Relative expression was calculated using the ΔΔCt method.

### 2.6 Western blotting

We isolated protein lysates from NSCLC cells via RIPA buffer (Cell Signaling Technology). The extract was centrifuged at 12,000 rpm at 4°C for 15 min after 10 min of vibration and then stored at −80°C. Sodium dodecyl sulfate–polyacrylamide gel electrophoresis was used to resolve the protein lysates. Subsequently, the samples were transferred to a nitrocellulose membrane (Millipore). After 2 h of transfer, the membranes were immersed in 5% BSA in Tris buffer saline with Tween 20 for 1 h and then immunoblotted with specific primary antibodies for more than 12 h at 4°C. The bands were immunoblotted with matched secondary antibodies for 2 h at 15–25°C on the second day. An enhanced chemiluminescence kit (Thermo Fisher) was used to visualize the bands. β‐Actin protein levels were used to normalize sample loading. The antibodies used in this research were as follows: anti‐FOXL2, anti‐TGF‐β receptor II, and anti‐MMP9 (Santa Cruz); anti‐TGF‐β receptor I (Abcam); anti‐p‐Smad3 (Ser423/425, C25A9), anti‐pAkt (Ser473, D9E), anti‐pErk (Thr202/Tyr204, D13.14.4 E), anti‐Smad3, anti‐Akt, anti‐Erk, anti‐CyclinD1, anti‐MMP2, anti‐Snail, and anti‐β‐actin (Cell Signaling Technology); anti‐N‐cadherin, anti‐E‐cadherin and anti‐Vimentin (BD Biosciences); and anti‐mouse and anti‐rabbit secondary antibodies (Cell Signaling Technology).

### 2.7 Cell proliferation analysis

NSCLC cell proliferation was evaluated by cell counting kit‐8 (CCK‐8) and colony formation assays. NSCLC cells were seeded in 96‐well plates at a density of 3 × 10^3^ cells per well and further grown in 96‐well plates under standard cell culture conditions. A CCK‐8 (Boster) was used to detect cell proliferation after culture for 24, 48, and 72 h. Colony formation assays were performed by seeding 60‐mm plates with 3000 cells per plate. After incubation for 7–14 days, according to the growth rate, colonies were stained with Giemsa and counted with ImageJ.

### 2.8 Wound healing assay

Wound healing assays were used to assess cell migration ability. NSCLC cells were cultured in 6‐well plates at 37°C until a monolayer was formed. Then, the monolayer was scratched in straight lines with a fresh 10‐μL pipette tip. Subsequently, sterile phosphate‐buffered saline (PBS) was used to wash the isolated cells, and then we refilled the complete medium in the wells. Cells were observed and imaged under a microscope (CKX41, Olympus) at the same magnification and settings after an additional 24 h.

### 2.9 Transwell assays

Cell migration and invasion abilities were assessed by Transwell migration and invasion assays. They were conducted using 8.0 μm Transwell inserts (Corning) with or without Matrigel (BD Science). For the migration assay, 3 × 10^4^ tumor cells in 200 μL RPMI‐1640 with 1% FBS were added into the upper chamber of the Transwell inserts. Then, 800 μL of RPMI‐1640 medium with 10% FBS was added to the lower chamber as an attractant. For the invasion assay, 5 × 10^4^ tumor cells diluted in 200 μL RPMI‐1640 with 1% FBS were seeded onto the upper chamber of a Transwell insert coated with Matrigel matrix (BD Science), and 800 μL RPMI‐1640 medium with 10% FBS was added to the lower chamber. If necessary, TGF‐β1 (5 ng/μL) was added to the lower chambers 6 h later. The Transwell system was then placed at 37°C for 24 h. After being washed with PBS twice, the Transwell inserts were fixed with methanol for 30 min and then air‐dried. The cells migrated to the lower surface and were stained with 0.1% crystal violet overnight afterward. Finally, the inserts were photographed and counted using a microscope (CKX41, Olympus).

### 2.10 Cell cycle analysis

According to the instructions of the Cell Cycle Analysis Kit (Beyotime), cells were cultured in 6‐well plates and were transfected with miR‐NC, miR‐133b, si‐NC, or si‐FOXL2 for 72 h. The cells were then collected, washed with cold PBS, fixed in 70% ethanol at 4°C for 24 h, and washed with cold PBS again. The cells were stained with a PI/RNase mixture from Beyotime Biotechnology, particularly in the dark, at 37°C for 30 min for the cell cycle analysis. Then, the stained cells were tested in a fluorescence‐activated cell sorting Caliber system (Beckman Coulter).

### 2.11 Luciferase reporter assay

To construct a plasmid containing the FOXL2 3′‐untranslated region (3′‐UTR) fused to the 3′‐end of a luciferase reporter, two fragments (217bp and 220 bp) involving the miR‐133b target sites (position 498–504, position 1261–1268) predicted by TargetScan were chosen for the luciferase assay. Two wild‐type and mutant fragments were directly synthesized (Genewiz) and fused to the 3′‐end of a luciferase reporter (psiCHECK2 dual‐luciferase vector; Promega). NSCLC cells were seeded in a 24‐well plate and cotransfected with the constructed plasmids and either miR‐133b mimics or miR‐NC using Lipofectamine 2000 (Life Technologies). The plates were maintained for 48 h, the cells were collected, and the luciferase activity was measured with the Dual‐Luciferase Reporter Assay kit (Promega).

### 2.12 Construction of a metastasis model in vivo

Female BALB/c athymic nude mice (4–6 weeks old and weighing 16–20 g) were purchased from the Experimental Animal Center of Soochow University and bred under pathogen‐free conditions. Sixteen mice were divided equally into two groups termed the FOXL2 silencing group and the control group (eight mice per group). A549 cells with FOXL2 knockdown (sh‐FOXL2) or stable negative control cells (sh‐NC) were intravenously (i.v.) injected (1 × 10^6^ cells per mouse) into the tail veins of the mice in the two groups. Mouse TGF‐β1 (4 mg/kg body weight) was intraperitoneally (i.p.) injected every 5 days after cell inoculation. The mice were sacrificed 50 days after inoculation, and their lung tissues were obtained and fixed in Bolin's fluid. The number of observable metastatic nodules on the surface of each lung was counted manually. Then, the tissues were histologically analyzed with H&E staining for the presence of micrometastases by H&E staining. All animal experiments were carried out according to the Guide for the Care and Use of Experimental Animals of the Experimental Animal Center of Soochow University.

### 2.13 Bioinformatics analysis

We conducted differential expression analysis, coexpression analysis, and KEGG pathway analysis using R Studio software, version 4.1.2. Data were downloaded from the TCGA online database (https://portal.gdc.cancer.gov), CCLE database (https://sites.broadinstitute.org/ccle/), or KOBAS (http://kobas.cbi.pku.edu.cn/). The Kaplan–Meier plot was produced by the Kaplan–Meier Plotter (http://www.kmplot.com). KEGG pathway analysis was conducted using gene set enrichment analysis 4.1.0 software. miRDB (https://www.mirdb.org/) and TargetScan Human (http://www.targetscan.org/) were used to identify the upstream regulator of the FOXL2 gene and the putative binding sites to the 3′‐UTR of FOXL2 mRNA for miR‐133b. We searched the online databases Oncomine (http://www.oncomine.org), STARBASE (https://starbase.sysu.edu.cn), GEPIA (http://gepia.cancer‐pku.cn), and UALCAN (http://ualcan.path.uab.edu/analysis.html) to conduct gene differential expression and RNA‐target or RNA‐RNA coexpression for supplements. Interactions between proteins were studied using the Search Tool for the Retrieval of Interacting Genes/Proteins (STRING) (https://string‐db.org).

### 2.14 Statistical analysis

Statistical analysis was performed using GraphPad Prism 7 software (GraphPad). All numerical data are presented as the mean ± standard deviation. We used Student's *t‐*test (two‐tailed) and two‐way ANOVA as appropriate to analyze the results. *p* < 0.05 was considered to indicate statistical significance.

## 3 RESULTS

### 3.1 FOXL2 is upregulated in NSCLC tissues and cell lines

**FIGURE 1:**
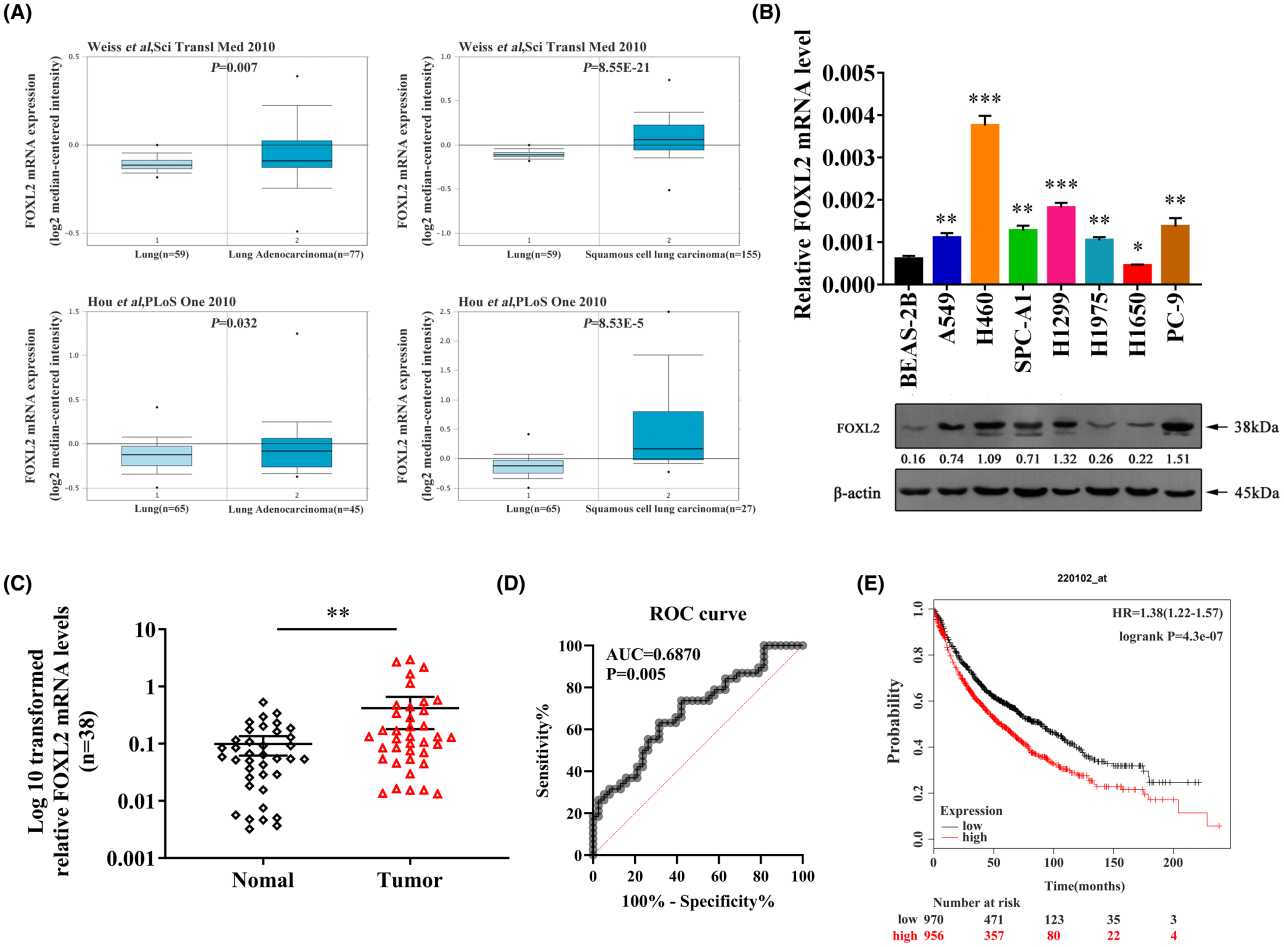
Forkhead box L2 (FOXL2) is upregulated in non‐small cell lung cancer (NSCLC) tissues and cell lines. (A) FOXL2 mRNA levels were markedly upregulated in NSCLC tissues. Data were obtained from the Oncomine online database (http://www.oncomine.org). (B) mRNA and protein levels of FOXL2 in cell lines measured by quantitative real‐time polymerase chain reaction (qRT–PCR) and western blotting. (C) FOXL2 mRNA levels in 38 paired NSCLC tissues and adjacent noncancerous lung tissues. (D) ROC curve of FOXL2 in 38 NSCLC tissues. AUC: area under curve. (E) Kaplan–Meier overall survival curve of NSCLC patients sorted based on FOXL2 expression. Kaplan–Meier plots were produced by Kaplan–Meier Plotter (http://www.kmplot.com). Bars represent the mean ± SD from three independent experiments. Significant differences compared with the control are indicated as follows: *p < 0.05; **p < 0.01; ***p < 0.001.

Gene differential expression analysis was conducted using data from the online databases Oncomine and TCGA. The results showed that FOXL2 mRNA levels were markedly increased in NSCLC tissues compared to control tissues (Figure 1A, Figure S1). Moreover, compared with the levels in the nonmetastatic group, FOXL2 expression levels were higher in the metastatic group (Figure S1). Consistently, we also found that FOXL2 was upregulated in NSCLC cell lines at both the mRNA and protein levels (Figure 1B). qRT–PCR was then carried out to evaluate FOXL2 mRNA levels in 38 paired NSCLC tissues. Increased FOXL2 levels were detected in the NSCLCs (28/38) (Figure 1C), whereas FOXL2 mRNA levels showed no significant correlation with the clinicopathological parameters except clinical stage (Table 1). We drew the ROC curve through the levels of 38 paired clinical samples. The area under the curve was 0.6870 and *p* < 0.05 (Figure 1D). We further analyzed the prognostic relevance of FOXL2 expression and overall survival (OS) in NSCLC. The Kaplan–Meier OS curve suggested that high FOXL2 expression predicted a poor prognosis (Figure 1E). Collectively, our data indicated that FOXL2 has good diagnostic efficacy and is expected to be an excellent index for the clinical NSCLC diagnosis and prognosis evaluation.

**TABLE 1:** Clinical characteristics and levels of FOXL2 mRNA expression in NSCLC tissues.

Characteristics	*n* = 38 (%)	FOXL2 mRNA expression		*p* value
		high	low	
		*n* = 28	*n* = 10	
*Age*				
≤65	21 (54)	17	4	0.2932
>65	17 (46)	11	6	
*Gender*				
Male	20 (52)	14	6	0.7190
Female	18 (48)	14	4	
*Histology*				
Adenocarcinoma	23 (60)	19	4	0.0742
Squamous cell carcinoma	9 (23)	4	5	
Others	6 (17)	5	1	
*Smoking status*				
Yes	16 (42)	9	7	0.0623
No	22 (58)	19	3	
*Clinical stage*				
Early	21 (54)	19	2	0.0171
Locally advanced	13(36)	6	7	
Advanced	4 (10)	3	1	
*Distant metastases*				
Yes	4 (10)	3	1	>0.9999
No	34 (90)	25	9	

*Note*: Data are presented as mean ± SD. Unpaired *t*‐test was used for comparison between two groups, and the Kruskal–Wallis test was used for comparison between three or more groups.

Abbreviations: FOXL2, Forkhead box L2; NSCLC, non‐small cell lung cancer.

### 3.2 Downregulation of FOXL2 suppresses cell proliferation and arrests the cell cycle in NSCLC

**FIGURE 2:**
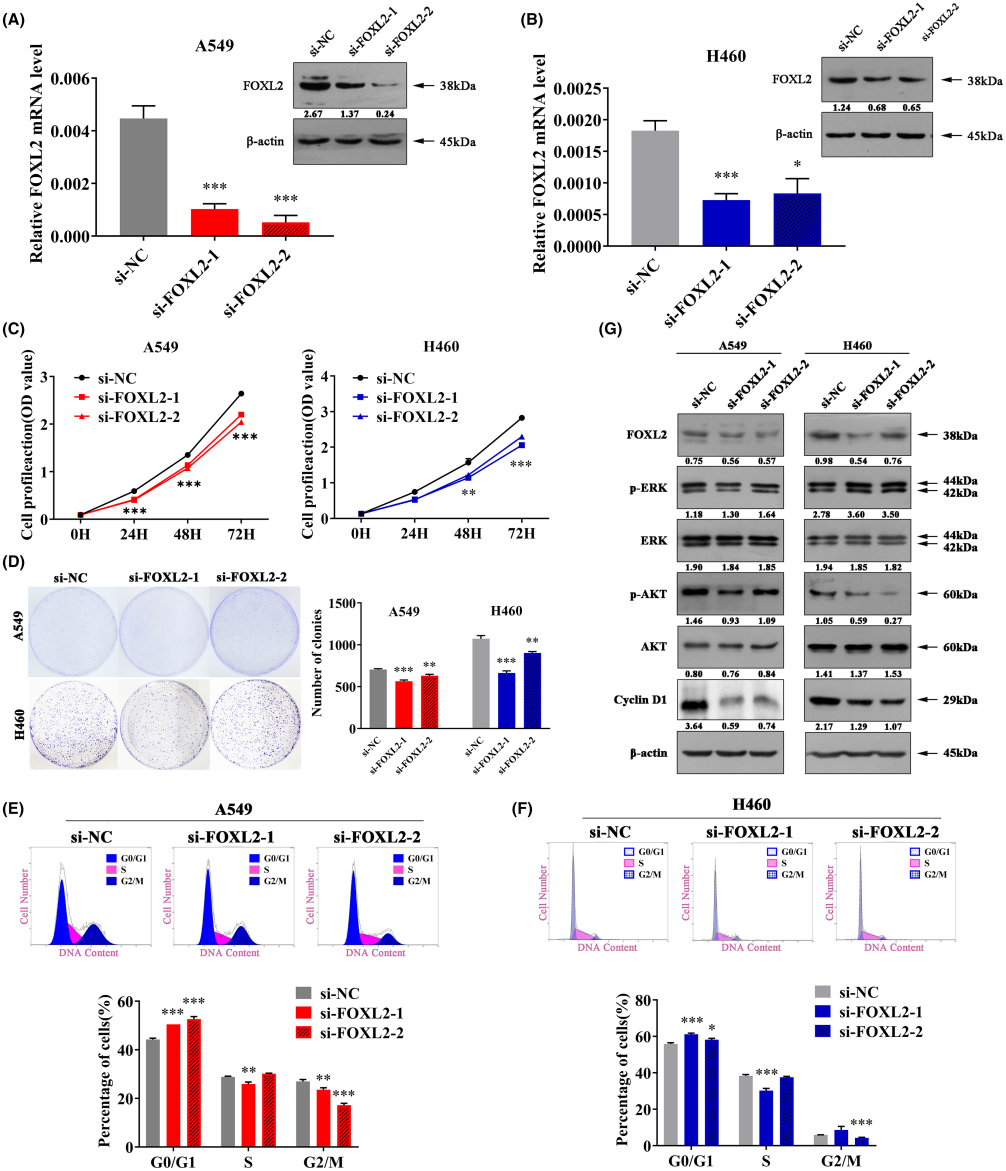
Downregulation of Forkhead box L2 (FOXL2) suppresses cell proliferation and arrests the cell cycle in non‐small cell lung cancer (NSCLC). (A, B) FOXL2 mRNA and protein levels in FOXL2 knockdown NSCLC cells. (C) Cell counting kit‐8 assays of cell viability in NSCLC cell lines. Cell viability was determined at 24, 48, and 72 h. (D) Clonogenic assays of cell proliferation in NSCLC cells. Bar charts show clonogenic growth of cells. (E, F) Flow cytometric analysis of NSCLC cell lines. Cells were harvested 72 h after transfection and stained with propidium iodide. The percentage of cells in each cell cycle phase is shown in the inset of each panel. (G) The levels of FOXL2, p‐Akt, Akt, p‐Erk, Erk, and Cyclin D1 were measured by western blotting. Bars represent the mean ± SD from three independent experiments. Significant differences compared with the control are indicated as follows: *p < 0.05; **p < 0.01; ***p < 0.001.

To clarify the effect of FOXL2 on the NSCLC cell phenotype, we constructed FOXL2 knockdown cell lines. Both FOXL2 RNA and protein levels were decreased in A549 and H460 cell lines (Figure 2A,B). CCK‐8 and clonogenic assays were applied to verify that FOXL2 knockdown can inhibit cell proliferation (Figure 2C,D). KEGG pathway analysis showed that FOXL2 may be essential to the cell cycle (Figure S2). We detected that number of cell in S phase was reduced, and the number of cells in G0/G1 phase was increased in FOXL2 knockdown cell lines compared to control cells (Figure 2E,F). Therefore, downregulation of FOXL2 can inhibit cell proliferation and arrest the cell cycle in NSCLC. The effect of FOXL2 on cell cycle progression may be due to its ability to regulate cell cycle mediators, such as Cyclin D1.^18^, ^52^ Western blotting further confirmed this finding (Figure 2G).

KEGG pathway analysis also showed that FOXL2 was associated with the PI3K/AKT signaling pathway (Figure S2). Western blot analysis showed that FOXL2 knockdown reduced the expression of the key components involved in PI3K‐AKT, such as p‐Akt, while p‐Erk was not decreased (Figure 2G). Overall, downregulation of FOXL2 inhibits cell proliferation, arrests the cell cycle and inactivates the PI3K/AKT signaling pathway in NSCLC.

### 3.3 Knockdown of FOXL2 inhibits cell migration and invasion via EMT in NSCLC

**FIGURE 3:**
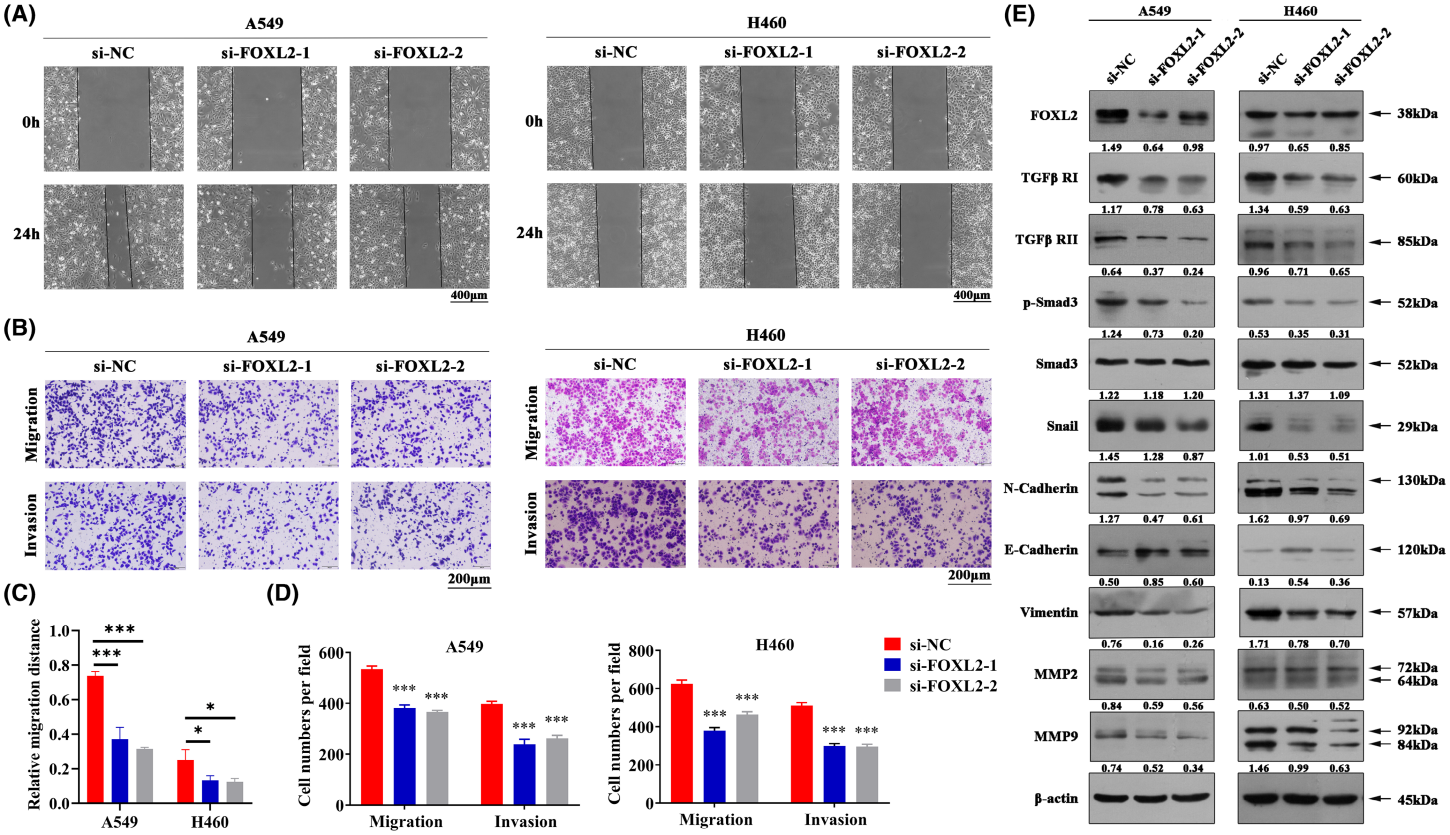
Knockdown of Forkhead box L2 (FOXL2) inhibits cell migration and invasion via epithelial–mesenchymal transition (EMT) in non‐small cell lung cancer (NSCLC). (A) Wound healing assays were performed to evaluate cell migration in NSCLC cells. Scale bar: 400 μm. (B) Representative images of the Transwell assays to evaluate cell migration and invasion in NSCLC cells. Scale bar: 200 μm. Bar charts show quantitative analyses of (C) wound healing assays and (D) Transwell assays. (E) The levels of FOXL2 and EMT‐related proteins were measured by western blotting in NSCLC cells. Bars represent the mean ± SD from three independent experiments. Significant differences compared with the control are indicated as follows: *p < 0.05; **p < 0.01; ***p < 0.001.

We further assessed the role of FOXL2 in regulating cell migration and invasion. Wound healing assays confirmed that the invasive ability was suppressed in both A549 and H460 cells after silencing FOXL2 (Figure 3A,C). Transwell assays showed that fewer cells migrated through the inserts in the presence of FOXL2 knockdown (Figure 3B,D). A previous publication reported that FOXL2 directly regulates the EMT process in chemoresistant gastric cancer.^28^ Coexpression analysis showed that FOXL2 was positively correlated with most of the genes involved in EMT (Figure S2). Additionally, KEGG pathway analysis implied that FOXL2 may play an important role in the TGF‐β signaling pathway (Figure S2). Therefore, we examined EMT‐related and TGF‐β signaling pathway‐related proteins to determine the underlying mechanism of FOXL2‐mediated metastasis in NSCLC. Western blot analysis showed that the expression levels of TGF‐β RI, TGF‐β RII, N‐cadherin, p‐Smad3, Snail, MMP2, MMP9, and Vimentin were markedly reduced after silencing FOXL2 (Figure 3E). Therefore, FOXL2 drives cell migration and invasion by inducing EMT.

### 3.4 FOXL2 induces EMT by activating Smad3 phosphorylation

**FIGURE 4:**
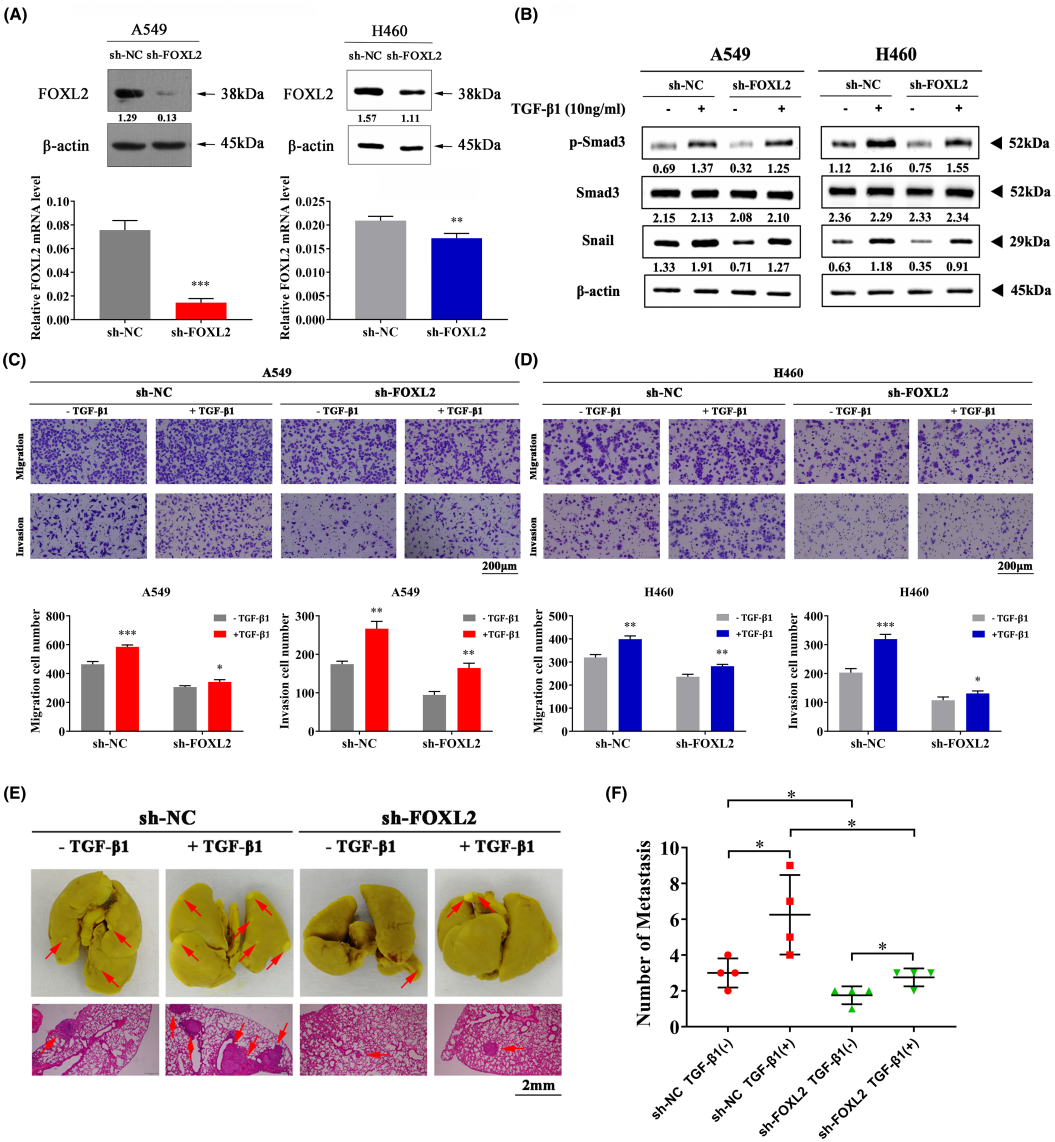
Forkhead box L2 (FOXL2) mediates the migration and invasion of non‐small cell lung cancer (NSCLC) cells through the transforming growth factor‐β (TGF‐β)/Smad signaling pathway. (A) FOXL2 mRNA and protein levels in NSCLC cells with stable FOXL2 knockdown. (B) After serum starvation for 24 h, NSCLC cells with stable FOXL2 knockdown were treated with TGF‐β1 (10 ng/mL) for 24 h. The expression of p‐Smad3, Smad3, and Snail was analyzed by western blotting. (C, D) Stable FOXL2‐knockdown NSCLC cells were treated with or without TGF‐β (10 ng/mL) for 24 h. Transwell assays were performed to evaluate cell migration and invasion in NSCLC cells. Scale bar: 200 μm. Bar charts show quantitative analyses of Transwell assays. (E, F) Representative images of pulmonary metastatic nodules and micrometastases detected by H&E staining are presented; scale bar: 2 mm, red arrowheads indicate micrometastases. Bars represent the mean ± SD from three independent experiments. Significant differences compared with the control are indicated as follows: *p < 0.05; **p < 0.01; ***p < 0.001.

It has been reported that the TGF‐β/Smad signaling pathway is crucial in the EMT process, and Smad3 is a key regulator of this signaling pathway.^36^, ^40^ To determine the underlying mechanism of FOXL2‐induced EMT, A549 and H460 cell lines were first chosen to construct stable FOXL2 knockdown cell lines. FOXL2 was significantly downregulated at both the mRNA and protein levels (Figure 4A). Next, we stimulated stable FOXL2 knockdown cell lines with exogenous TGF‐β1 at a concentration of 10 ng/mL. Western blotting showed that TGF‐β1‐induced upregulation of Smad3 phosphorylation and Snail was attenuated in FOXL2 knockdown cells (Figure 4B). Moreover, Transwell assays implied that promotion of cell migration and invasion induced by TGF‐β1 was attenuated by the knockdown of FOXL2 (Figure 4C,D). Therefore, FOXL2 induces EMT by stimulating the TGF‐β/Smad signaling pathway.

### 3.5 Downregulation of FOXL2 inhibits cell metastasis in vivo

To determine the effects of FOXL2 downregulation on cell metastasis in vivo, we intravenously injected sh‐FOXL2 or sh‐NC A549 cells into BALB/c athymic nude mice. Mice were injected with TGF‐β1 intraperitoneally after cell inoculation. We dissected lung tissues, fixed them and used Bouin's fluid to stain the tissues. Obvious pulmonary metastatic nodules were visible in the stable FOXL2 knockdown group and the NC group (Figure 4E,F, Figure S3). Fewer pulmonary metastatic nodules and pulmonary micrometastases were detected in the mice injected with A549 cells with stable FOXL2 knockdown than in the NC mice (Figure 4E,F). Additionally, knockdown of FOXL2 suppressed TGF‐β1‐induced A549 cell metastasis (Figure 4E,F). Collectively, these findings indicate that FOXL2 knockdown inhibited cell metastasis in vivo.

### 3.6 MiR‐133b is downregulated in NSCLC and targets FOXL2 directly

**FIGURE 5:**
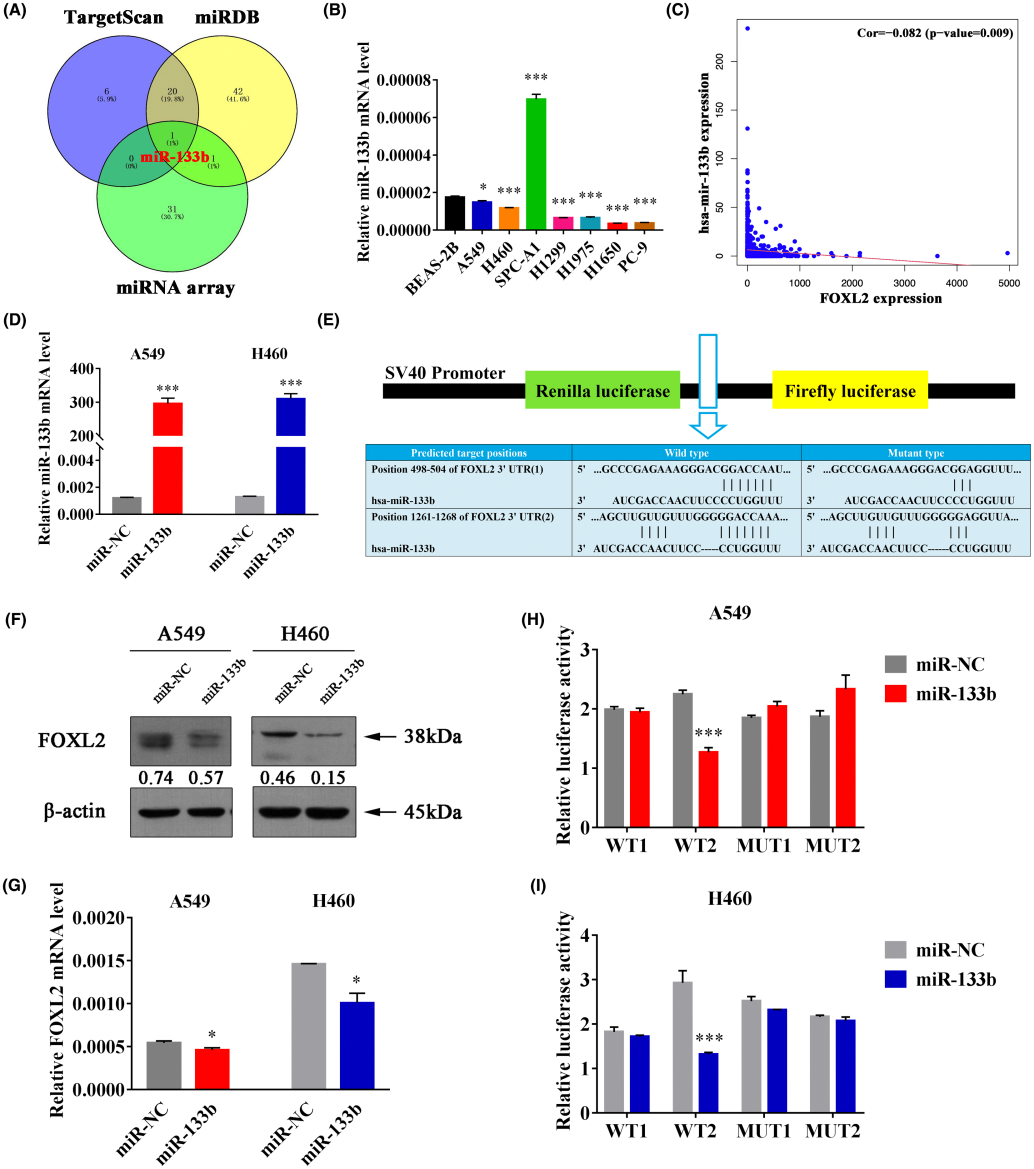
MiR‐133b is downregulated in non‐small cell lung cancer (NSCLC) and targets Forkhead box L2 (FOXL2) directly. (A) Venn diagram showing that miR‐133b was identified as a potential upstream regulator of FOXL2. (B) The level of miR‐133b in cell lines measured quantitative real‐time polymerase chain reaction (qRT–PCR). (C) Coexpression analysis showed that FOXL2 and miR‐133b were negatively correlated in NSCLC patient samples in the TCGA database. (D) The level of miR‐133b was increased when NSCLC cells were transfected with miR‐133b mimics. (E) Schematic diagram showing the subcloning of the predicted miR‐133b binding site at two positions (498–504 and 1261–1268) of the FOXL2 3′‐UTR into a psiCHECK‐2 luciferase construct. The predicted duplex formed between miR‐133b and the wild‐type or mutant miR‐133b binding site is indicated. (F, G) FOXL2 mRNA and protein levels were decreased in miR‐133b‐overexpressing NSCLC cells. (H, I) Luciferase activity of the construct containing the wild‐type or mutant FOXL2 3′‐UTR reporter gene in A549 and H460 cells cotransfected with the negative control (NC) or miR‐133b. Scrambled sequences were used as the NC. Relative Renilla luciferase activity was determined and normalized against firefly luciferase activity. Bars represent the mean ± SD from three independent experiments. Significant differences compared with the control are indicated as follows: *p < 0.05; **p < 0.01; ***p < 0.001.

Since the expression regulation mechanism of FOXL2 remains unknown and miRNAs can directly regulate gene expression,^53^, ^54^ we aimed to identify miRNAs targeting FOXL2 in this study. By overlapping the miRNAs predicted in the online biological databases miRDB (https://www.mirdb.org/) and TargetScan Human (http://www.targetscan.org/), as well as the downregulated miRNAs in the miRNA microarray of four paired NSCLC and normal tissues, we identified miR‐133b as a potential upstream regulator of FOXL2 (Figure 5A). Bioinformatics analysis showed that miR‐133b was significantly downregulated in NSCLC tissues (Figure S4). We then quantified the expression level of miR‐133b in cell lines, and confirmed that most NSCLC cell lines showed lower miR‐133b expression than BEAS‐2B cells (Figure 5B). Coexpression analysis showed that FOXL2 and miR‐133b were negatively correlated in NSCLC patient samples in the TCGA database (https://portal.gdc.cancer.gov) (Figure 5C, Figure S4). Thus, it is highly likely that miR‐133b negatively regulates FOXL2 expression. To confirm that FOXL2 expression is directly regulated by miR‐133b, we transfected NSCLC cell lines with miR‐133b mimics to upregulate miR‐133b (Figure 5D). As expected, miR‐133b overexpression significantly suppressed the mRNA and protein expression of FOXL2 (Figure 5F,G). Further analysis via the online database TargetScan Human (http://www.targetscan.org/) identified two putative binding sites for miR‐133b on the 3′‐UTR of FOXL2 mRNA (Figure 5E). We then subcloned the FOXL2 3′‐UTR containing the miR‐133b binding site (WT1, WT2/MUT1, MUT2) into the psiCHECK‐2 vector, followed by dual‐luciferase reporter assays. The results showed that miR‐133b significantly decreased luciferase activity in cells transfected with the WT2 FOXL2 3′‐UTR but did not decrease luciferase activity in cells containing the WT1, MUT1 or MUT2 constructs (Figure 5H,I). Hence, predicted binding site 2 was verified as a valid binding site. Collectively, these findings indicate that miR‐133b directly targets and negatively regulates FOXL2.

### 3.7 MiR‐133b overexpression inhibits cell proliferation, migration, and invasion and arrests the cell cycle in NSCLC

**FIGURE 6:**
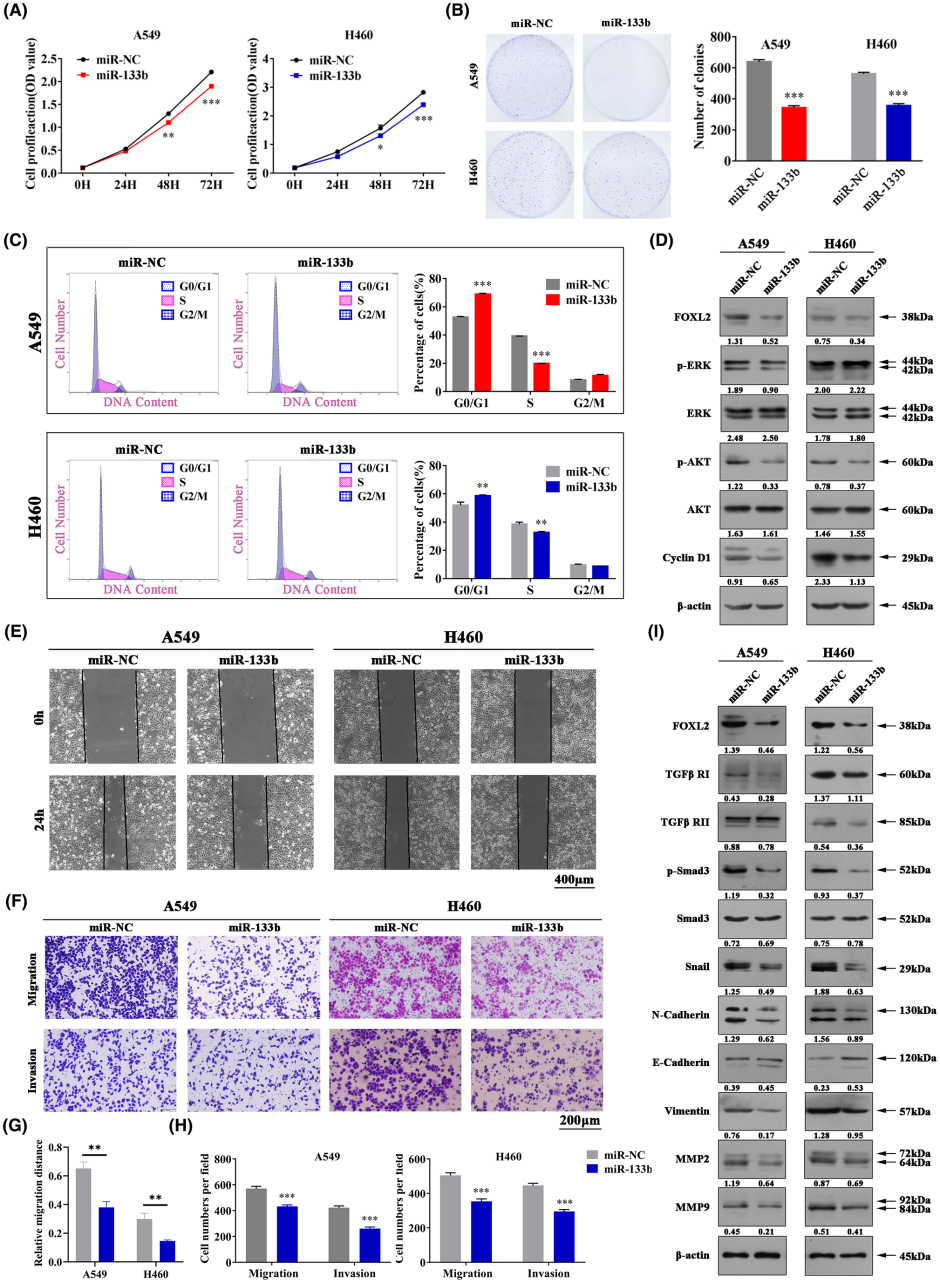
MiR‐133b overexpression inhibits cell proliferation, migration, and invasion and arrests the cell cycle in non‐small cell lung cancer (NSCLC). (A) Cell counting kit‐8 assays of cell viability in NSCLC cell lines. Cell viability was determined at 24, 48, and 72 h. (B) Clonogenic assays of cell proliferation in NSCLC cells. Bar charts show clonogenic growth of cells. (C) Flow cytometric analysis of NSCLC cell lines. Cells were harvested at 72 h after transfection and stained with propidium iodide. The percentage of cells in each cell cycle phase is shown in the inset of each panel. (D) The levels of Forkhead box L2 (FOXL2), p‐Akt, Akt, p‐Erk, Erk, and Cyclin D1 were measured by western blotting. (E) Wound healing assays were performed to evaluate cell migration in NSCLC cells. Scale bar: 400 μm. (F) Representative images of the Transwell assays to evaluate cell migration and invasion in NSCLC cells. Scale bar: 200 μm. Bar charts show quantitative analyses of (G) wound healing assays and (H) Transwell assays. (I) The levels of FOXL2 and epithelial–mesenchymal transition (EMT)‐related proteins were measured by western blotting in NSCLC cells. Bars represent the mean ± SD from three independent experiments. Significant differences compared with the control are indicated as follows: *p < 0.05; **p < 0.01; ***p < 0.001.

We further determined the function of miR‐133b in NSCLC. CCK‐8 and clonogenic assays showed that NSCLC cells overexpressing miR‐133b had significantly lower proliferation ability than the control cells (Figure 6A,B). The proportion of cells in the G0/G1 phase was efficiently higher and in the S phase was lower in NSCLC cells in miR‐133b‐overexpressing NSCLC cells by the flow cytometry (Figure 6C). Moreover, wound healing and Transwell assays showed that miR‐133b significantly attenuated cell migration and invasion in NSCLC (Figure 6E–H). Mechanistically, when compared to the control group, the protein levels of FOXL2 and its downstream signaling molecules, such as p‐Akt, Cyclin D1, TGF‐β RI, TGF‐β RII, p‐Smad3, Snail, N‐cadherin, Vimentin, MMP2, and MMP9, were obviously decreased in cells transfected with miR‐133b mimics (Figure 6D,I), which was in line with the results in the FOXL2‐knockdown cells (Figures 2G and 3E). Collectively, these results show that miR‐133b inhibits cell proliferation, migration, and invasion and arrests the cell cycle by negatively regulating FOXL2.

### 3.8 Downregulation of miR‐133b reverses the inhibition of cell proliferation, migration and invasion induced by FOXL2 knockdown

**FIGURE 7:**
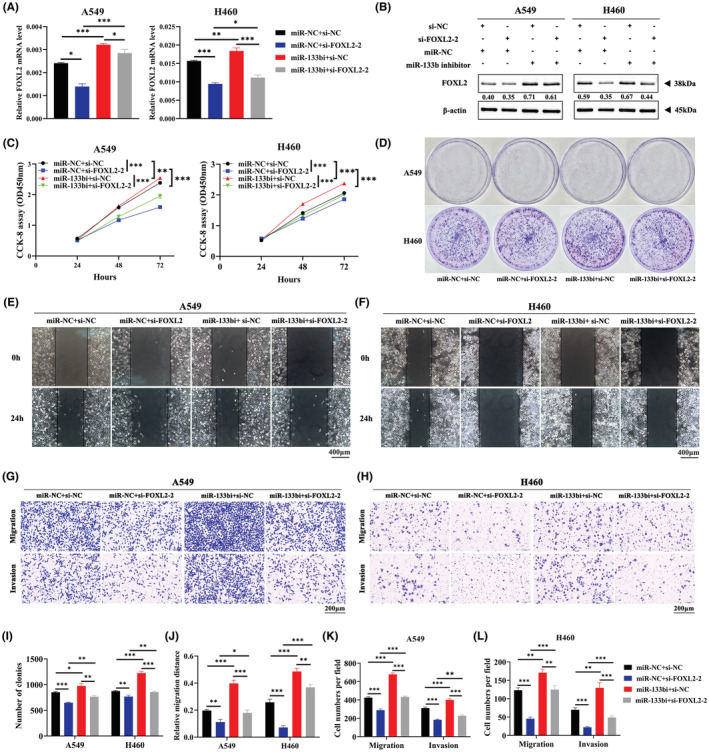
Downregulation of miR‐133b reverses the inhibition of cell proliferation, migration, and invasion induced by FOXL2 knockdown. (A, B) Forkhead box L2 (FOXL2) mRNA and protein levels in non‐small cell lung cancer (NSCLC) cells. (C) Cell counting kit‐8 (CCK‐8) assays of cell viability in NSCLC cell lines. Cell viability was determined at 24, 48, and 72 h. (D) Clonogenic assays of cell proliferation in NSCLC cells. (E, F) Wound healing assay was performed to evaluate cell migration in NSCLC cells. Scale bar: 400 μm. (G, H) Representative images of the Transwell assays to evaluate cell migration and invasion in NSCLC cells. Scale bar: 200 μm. (I) Bar charts show clonogenic growth of cells. (J) Bar charts show quantitative analyses of wound healing assays. (K, L) Bar charts showed quantitative analyses of Transwell assays. miR‐133bi: miR‐133b inhibitor. Bars represent the mean ± SD from three independent experiments. Significant differences compared with the control are indicated as follows: *p < 0.05; **p < 0.01; ***p < 0.001.

To gain deeper insight into the influence of the miR‐133b/FOXL2 axis in NSCLC, we performed rescue experiments in NSCLC cells. We transfected NSCLC cells with miR‐133b inhibitor and si‐FOXL2 and cotransfected miR‐NC and si‐NC as controls. The mRNA and protein levels of FOXL2 are shown in Figure 7A,B. The results implied that cell proliferation was inhibited by knockdown of FOXL2 cells, while this negative effect was reversed by inhibition of miR‐133b in CCK‐8 and clonogenic assays (Figure 7C,D,I). Wound healing assays and Transwell assays revealed that the inhibition of cell migration and invasion induced by FOXL2 knockdown was reversed by the miR‐133b inhibitor (Figure 7E–H,J–L). Therefore, inhibition of miR‐133b reverses the suppressive effect of FOXL2 knockdown on cell proliferation, migration, and invasion.

## 4 DISCUSSION

Tumor cells are characterized by malignant proliferation, migration, and invasion, which significantly affect the development of cancer. However, the exact mechanism remains to be fully elucidated, especially in lung cancer, which we focused on. Aberrant gene expression^55^ and epigenetic modifications, have been proven to be involved in tumorigenesis. Moreover, the microenvironment in which cancer cells develop and survive is critical to tumor progression and treatment responese.^56^ Many attempts have been made to investigate the initiation and progression of NSCLC. Guo et al. developed the GILncSig as an independent marker with prognostic value by combining lncRNA expression profiles with clinical features of lung adenocarcinoma (LUAD) and somatic mutation profiles.^57^ Additionally, understanding a single tumor's epigenetic modification alteration pattern can improve our knowledge of the tumor microenvironment and guide the treatment of LUAD.^56^ In the current study, we reported that miR‐133b downregulated FOXL2 by targeting its 3′‐UTR, thereby inhibiting cell proliferation, EMT and metastasis induced by the TGF‐β/Smad signaling pathway in NSCLC. FOXL2 may be a potential molecular target for treating NSCLC.

FOXL2 encodes a transcription factor that was initially found to play an important role in sex determination, ovarian maintenance and function, and genomic maintenance.^58^ FOXL2 can regulate cellular pathways, including cell cycle progression, proliferation, and apoptosis.^58^ Studies have increasingly found that FOXL2 participates in tumorigenesis. FOXL2 participates in the occurrence and development of numerous tumors, such as ovarian GCT^27^ and chem‐resistant gastric cancer.^28^, ^29^ However, the function of FOXL2 in NSCLC has not yet been identified. In the current study, we confirmed that FOXL2 is upregulated in NSCLC, and can promote cell proliferation, migration, and invasion, and regulate the cell cycle in NSCLC. FOXL2 has good diagnostic efficacy and is expected to be an excellent index for the clinical NSCLC diagnosis and prognosis evaluation.

We next sought to elucidate the specific mechanism underlying the FOXL2‐induced oncogenic effects in NSCLC. In this study, we found that FOXL2 knockdown inhibited cell proliferation and arrested the cell cycle. The effect of FOXL2 on cell cycle progression may be due to its ability to regulate cell cycle mediators, such as Cyclin D1.^18^, ^52^ Western blotting further confirmed this conclusion. We assumed that the number of NSCLC cells in the DNA synthesis phase decreased after FOXL2 interference, which led to the suppression of cell proliferation. Moreover, bioinformatics analysis showed that FOXL2 is related to the PI3K/AKT signaling pathway, and western blot analysis showed that FOXL2 knockdown reduced the expression of p‐Akt, a key component involved in PI3K/AKT. It has been suggested that FOX factors are key regulators in the PI3K/AKT signaling pathway that participate in regulating the PI3K/AKT molecular cascade.^59^ It has also been shown that AKT activation induces FOXO3 accumulation in the cytoplasm via 14‐3‐3 protein action.^60^, ^61^ We hypothesized that there exists a feedback regulatory mechanism between FOXL2 and AKT in NSCLC proliferation. Further studies are needed to verify our conjectures.

Metastasis is also a critical issue leading to poor survival in NSCLC, and EMT plays a key role in tumor metastasis by promoting cell migration, invasion and metastasis.^39^ The TGF‐β/Smad signaling pathway is critical in the EMT process of various types of epithelial cells, and Smad3 is a key regulator in this pathway.^36^, ^40^ Studies have focused on how to stop the progression of cancer by reversing or delaying EMT. A previous study discovered that FOXL2 was abnormally high in gastric carcinoma and promoted EMT‐induced metastasis in chem‐resistant gastric cancer.^28^, ^29^ In the current study, we found a positive correlation between FOXL2 and most of the genes involved in EMT. Knockdown of FOXL2 in NSCLC significantly decreased TGF‐β receptor I, TGF‐β receptor II, Snail, p‐Smad3, N‐cadherin, MMP2, MMP9, and Vimentin levels and markedly increased E‐cadherin expression, and the metastatic ability of NSCLC cells was reduced. Adding TGF‐β1 promotes the migration and invasion of FOXL2 knockdown cells and increased the expression of p‐Smad3 and its downstream signaling molecules. These results suggested that FOXL2 can induce EMT by activating Smad3 phosphorylation. However, the detailed mechanism by which FOXL2 affects the TGF‐β/Smad signaling pathways needed further investigation. Secchi et al. suggested that the FOXL2 mutant may drive the steady induction of its key molecular Smad partners via a direct TGF‐β2 positive feedback loop.^62^ Anttonen et al.^63^ found that the interaction between FOXL2, GATA4, and Smad3 modulates the promoter activity of the key target genes involved in GCT cell proliferation and survival, such as CCND2. FOXL2 was predicted to interact with Smad via the STRING online database (https://string‐db.org) (Figure S2). Hence, we hypothesized that FOXL2 could interact with Smad and modulate NSCLC cell viability, which may be the specific molecular mechanism of FOXL2 in NSCLC tumorigenesis. We will confirm the hypothesis in our further work.

Since there is ample evidence for the oncogenic role of FOXL2 in NSCLC, we next identified the expression regulation mechanism of FOXL2. We searched the online database and identified miR‐133b as a putative upstream regulator of the FOXL2 gene, as well as the two putative binding sites for miR‐133b on the 3′‐UTR of FOXL2 mRNA. Our previous study also showed that the miR‐133b was downregulated in lung cancer tissues.^50^ Coexpression analysis and dual‐luciferase reporter assays further confirmed that miR‐133b directly targeted FOXL2 through binding site‐2 and negatively regulated FOXL2. We revealed that miR‐133b was downregulated in NSCLC, which was consistent with previous findings in other cancer types.^47^, ^48^, ^49^ Overexpression of miR‐133b could suppress the proliferation, migration, and invasion of ESCC cells by inhibiting the MAPK/ERK and PI3K/AKT signaling pathways through targeting EGFR.^64^ Our study verified that miR‐133b inhibited cell proliferation, migration, and invasion by suppressing the PI3K/AKT and TGF‐β/Smad signaling pathways in NSCLC. miR‐133b was also proven to inhibit TGF‐β1‐induced EMT and renal fibrosis by upregulating SIRT1 in diabetic nephropathy.^65^ Rescue experiments were conducted to further confirm the negative regulation of FOXL2 by miR‐133b.

## 5 CONCLUSION

Generally, miR‐133b downregulates FOXL2 by targeting the 3′‐UTR of FOXL2, thereby inhibiting cell proliferation, EMT, and metastasis induced by the TGF‐β/Smad signaling pathway in NSCLC. FOXL2 may be a potential molecular target for NSCLC treatment.

## AUTHOR CONTRIBUTIONS

**Juan Li:** Methodology (equal); software (equal); validation (equal); writing – original draft (equal). **Lirong Gao:** Methodology (equal); software (equal); validation (equal); writing – original draft (equal). **Anqi Wang:** Methodology (equal); validation (equal); writing – original draft (equal). **Huiwen Qian:** Investigation (supporting); software (supporting); visualization (supporting). **Jianjie Zhu:** Methodology (supporting); validation (supporting); visualization (supporting). **Shundong Ji:** Methodology (supporting); resources (supporting). **Jun Chen:** Resources (equal); supervision (equal); writing – original draft (equal). **Zeyi Liu:** Conceptualization (equal); formal analysis (equal); funding acquisition (equal); project administration (equal); writing – review and editing (equal). **Cheng Ji:** Conceptualization (equal); formal analysis (equal); funding acquisition (equal); project administration (equal); writing – review and editing (equal).

## FUNDING INFORMATION

The research was funded by grants from Suzhou Science and Technology Plan Project (SLJ202003) and The Suzhou Gusu Medical Youth Talent (GSWS2020016).

## CONFLICT OF INTEREST STATEMENT

The authors declare no conflict of interest.

## ETHICS STATEMENT

The study was approved by the Ethics Committee of the First Affiliated Hospital of Soochow University. The corresponding ethical approval code is 2020‐375.

## Supporting information


Figure S1.

Figure S2.

Figure S3.

Figure S4.

## Data Availability

All original data and the datasets generated for this study are available on request to the corresponding author.

## References

[1] Sung H , Ferlay J , Siegel RL , et al. Global cancer statistics 2020: GLOBOCAN estimates of incidence and mortality worldwide for 36 cancers in 185 countries. CA Cancer J Clin. 2021;71(3):209‐249. 33538338 10.3322/caac.21660

[2] Reck M , Rabe KF . Precision diagnosis and treatment for advanced non‐small‐cell lung cancer. N Engl J Med. 2017;377(9):849‐861. 28854088 10.1056/NEJMra1703413

[3] Chen R , Xu X , Qian Z , et al. The biological functions and clinical applications of exosomes in lung cancer. Cell Mol Life Sci. 2019;76(23):4613‐4633. 31352532 10.1007/s00018-019-03233-y PMC11105651

[4] Arbour KC , Riely GJ . Systemic therapy for locally advanced and metastatic non‐small cell lung cancer: a review. JAMA. 2019;322(8):764‐774. 31454018 10.1001/jama.2019.11058

[5] Siegel RL , Miller KD , Fuchs HE , Jemal A . Cancer statistics, 2022. CA Cancer J Clin. 2022;72(1):7‐33. 35020204 10.3322/caac.21708

[6] Miller KD , Nogueira L , Mariotto AB , et al. Cancer treatment and survivorship statistics, 2019. CA Cancer J Clin. 2019;69(5):363‐385. 31184787 10.3322/caac.21565

[7] Westover D , Zugazagoitia J , Cho BC , Lovly CM , Paz‐Ares L . Mechanisms of acquired resistance to first‐ and second‐generation EGFR tyrosine kinase inhibitors. Ann Oncol. 2018;29(suppl_1):i10‐i19. 29462254 10.1093/annonc/mdx703 PMC6454547

[8] Saito H , Fukuhara T , Furuya N , et al. Erlotinib plus bevacizumab versus erlotinib alone in patients with EGFR‐positive advanced non‐squamous non‐small‐cell lung cancer (NEJ026): interim analysis of an open‐label, randomised, multicentre, phase 3 trial. Lancet Oncol. 2019;20(5):625‐635. 30975627 10.1016/S1470-2045(19)30035-X

[9] Iams WT , Porter J , Horn L . Immunotherapeutic approaches for small‐cell lung cancer. Nat Rev Clin Oncol. 2020;17(5):300‐312. 32055013 10.1038/s41571-019-0316-z PMC7212527

[10] Cheng H , Li XJ , Wang XJ , et al. A meta‐analysis of adjuvant EGFR‐TKIs for patients with resected non‐small cell lung cancer. Lung Cancer. 2019;137:7‐13. 31520922 10.1016/j.lungcan.2019.08.002

[11] Lee DH . Treatments for EGFR‐mutant non‐small cell lung cancer (NSCLC): the road to a success, paved with failures. Pharmacol Ther. 2017;174:1‐21. 28167215 10.1016/j.pharmthera.2017.02.001

[12] Sui H , Ma N , Wang Y , et al. Ant‐PD‐1/PD‐L1 therapy for non‐small‐cell lung cancer: toward personalized medicine and combination strategies. J Immunol Res. 2018;2018:6984948. 30159341 10.1155/2018/6984948 PMC6109480

[13] Borghaei H , Gettinger S , Vokes EE , et al. Five‐year outcomes from the randomized, phase III trials CheckMate 017 and 057: nivolumab versus docetaxel in previously treated non‐small‐cell lung cancer. J Clin Oncol. 2021;39(7):723‐733. 33449799 10.1200/JCO.20.01605 PMC8078445

[14] Gurnari C , Falconi G , De Bellis E , Voso MT , Fabiani E . The role of forkhead box proteins in acute myeloid leukemia. Cancers (Basel). 2019;11(6):865‐883. 31234353 10.3390/cancers11060865 PMC6627614

[15] Wang J , Li W , Zhao Y , et al. Members of FOX family could be drug targets of cancers. Pharmacol Ther. 2018;181:183‐196. 28830838 10.1016/j.pharmthera.2017.08.003

[16] Gong Z , Yu J , Yang S , Lai PBS , Chen GG . FOX transcription factor family in hepatocellular carcinoma. Biochim Biophys Acta Rev Cancer. 2020;1874(1):188376. 32437734 10.1016/j.bbcan.2020.188376

[17] Lo PK , Lee JS , Liang X , Sukumar S . The dual role of FOXF2 in regulation of DNA replication and the epithelial‐mesenchymal transition in breast cancer progression. Cell Signal. 2016;28(10):1502‐1519. 27377963 10.1016/j.cellsig.2016.06.021 PMC5056599

[18] Tucker EJ . The genetics and biology of FOXL2. Sex Dev. 2022;16(2‐3):184‐193. 34727551 10.1159/000519836

[19] Golson ML , Kaestner KH . Fox transcription factors: from development to disease. Development. 2016;143(24):4558‐4570. 27965437 10.1242/dev.112672 PMC5201025

[20] Cocquet J , De Baere E , Gareil M , et al. Structure, evolution and expression of the FOXL2 transcription unit. Cytogenet Genome Res. 2003;101(3–4):206‐211. 14684984 10.1159/000074338

[21] Georges A , Auguste A , Bessière L , Vanet A , Todeschini AL , Veitia RA . FOXL2: a central transcription factor of the ovary. J Mol Endocrinol. 2014;52(1):R17‐R33. 24049064 10.1530/JME-13-0159

[22] Ellsworth BS , Egashira N , Haller JL , et al. FOXL2 in the pituitary: molecular, genetic, and developmental analysis. Mol Endocrinol. 2006;20(11):2796‐2805. 16840539 10.1210/me.2005-0303

[23] Rosario R , Cohen PA , Shelling AN . The role of FOXL2 in the pathogenesis of adult ovarian granulosa cell tumours. Gynecol Oncol. 2014;133(2):382‐387. 24342437 10.1016/j.ygyno.2013.12.012

[24] Duffin K , Bayne RA , Childs AJ , Collins C , Anderson RA . The forkhead transcription factor FOXL2 is expressed in somatic cells of the human ovary prior to follicle formation. Mol Hum Reprod. 2009;15(12):771‐777. 19706741 10.1093/molehr/gap065 PMC2776473

[25] Crisponi L , Deiana M , Loi A , et al. The putative forkhead transcription factor FOXL2 is mutated in blepharophimosis/ptosis/epicanthus inversus syndrome. Nat Genet. 2001;27(2):159‐166. 11175783 10.1038/84781

[26] Rosario R , Araki H , Print CG , Shelling AN . The transcriptional targets of mutant FOXL2 in granulosa cell tumours. PLoS ONE. 2012;7(9):e46270. 23029457 10.1371/journal.pone.0046270 PMC3460904

[27] Leung DTH , Fuller PJ , Chu S . Impact of FOXL2 mutations on signaling in ovarian granulosa cell tumors. Int J Biochem Cell Biol. 2016;72:51‐54. 26791928 10.1016/j.biocel.2016.01.003

[28] Dong J , Wang R , Ren G , et al. HMGA2‐FOXL2 axis regulates metastases and epithelial‐to‐mesenchymal transition of chemoresistant gastric cancer. Clin Cancer Res. 2017;23(13):3461‐3473. 28119367 10.1158/1078-0432.CCR-16-2180

[29] Yu L , Chen J , Liu Y , Zhang Z , Duan S . MicroRNA‐937 inhibits cell proliferation and metastasis in gastric cancer cells by downregulating FOXL2. Cancer Biomark. 2017;21(1):105‐116. 29060929 10.3233/CBM-170310 PMC13075738

[30] Zhao Y , Zhou H , Ma K , et al. Abnormal methylation of seven genes and their associations with clinical characteristics in early stage non‐small cell lung cancer. Oncol Lett. 2013;5(4):1211‐1218. 23599765 10.3892/ol.2013.1161 PMC3629069

[31] Kuang S , Fung AS , Perdrizet KA , et al. Upfront next generation sequencing in non‐small cell lung cancer. Curr Oncol. 2022;29(7):4428‐4437. 35877212 10.3390/curroncol29070352 PMC9319994

[32] Singh M , Yelle N , Venugopal C , Singh SK . EMT: mechanisms and therapeutic implications. Pharmacol Ther. 2018;182:80‐94. 28834698 10.1016/j.pharmthera.2017.08.009

[33] Mitschke J , Burk UC , Reinheckel T . The role of proteases in epithelial‐to‐mesenchymal cell transitions in cancer. Cancer Metastasis Rev. 2019;38(3):431‐444. 31482486 10.1007/s10555-019-09808-2

[34] Nieto MA , Huang RY , Jackson RA , Thiery JP . EMT: 2016. Cell. 2016;166(1):21‐45. 27368099 10.1016/j.cell.2016.06.028

[35] Zeisberg M , Neilson EG . Biomarkers for epithelial‐mesenchymal transitions. J Clin Invest. 2009;119(6):1429‐1437. 19487819 10.1172/JCI36183 PMC2689132

[36] Dongre A , Weinberg RA . New insights into the mechanisms of epithelial–mesenchymal transition and implications for cancer. Nat Rev Mol Cell Biol. 2018;20(2):69‐84. 10.1038/s41580-018-0080-4 30459476

[37] Nakaya Y , Sheng G . EMT in developmental morphogenesis. Cancer Lett. 2013;341(1):9‐15. 23462225 10.1016/j.canlet.2013.02.037

[38] Campbell K . Contribution of epithelial‐mesenchymal transitions to organogenesis and cancer metastasis. Curr Opin Cell Biol. 2018;55:30‐35. 30006053 10.1016/j.ceb.2018.06.008 PMC6284102

[39] Feng YL , Chen DQ , Vaziri ND , Guo Y , Zhao YY . Small molecule inhibitors of epithelial‐mesenchymal transition for the treatment of cancer and fibrosis. Med Res Rev. 2020;40(1):54‐78. 31131921 10.1002/med.21596

[40] Suriyamurthy S , Baker D , Ten Dijke P , Iyengar PV . Epigenetic reprogramming of TGF‐beta signaling in breast cancer. Cancers (Basel). 2019;11(5):726‐738. 31137748 10.3390/cancers11050726 PMC6563130

[41] Zhang P , Tang WM , Zhang H , et al. MiR‐646 inhibited cell proliferation and EMT‐induced metastasis by targeting FOXK1 in gastric cancer. Br J Cancer. 2017;117(4):525‐534. 28632723 10.1038/bjc.2017.181 PMC5558677

[42] Zhang X , Zhang L , Du Y , et al. A novel FOXM1 isoform, FOXM1D, promotes epithelial‐mesenchymal transition and metastasis through ROCKs activation in colorectal cancer. Oncogene. 2017;36(6):807‐819. 27399334 10.1038/onc.2016.249 PMC5311249

[43] Treiber T , Treiber N , Meister G . Regulation of microRNA biogenesis and its crosstalk with other cellular pathways. Nat Rev Mol Cell Biol. 2019;20(1):5‐20. 30228348 10.1038/s41580-018-0059-1

[44] Jonas S , Izaurralde E . Towards a molecular understanding of microRNA‐mediated gene silencing. Nat Rev Genet. 2015;16(7):421‐433. 26077373 10.1038/nrg3965

[45] Pasquinelli AE . MicroRNAs and their targets: recognition, regulation and an emerging reciprocal relationship. Nat Rev Genet. 2012;13(4):271‐282. 22411466 10.1038/nrg3162

[46] Cai Y , Yu X , Hu S , Yu J . A brief review on the mechanisms of miRNA regulation. Genom Proteom Bioinform. 2009;7(4):147‐154. 10.1016/S1672-0229(08)60044-3 PMC5054406 20172487

[47] Wang QY , Zhou CX , Zhan MN , et al. MiR‐133b targets Sox9 to control pathogenesis and metastasis of breast cancer. Cell Death Dis. 2018;9(7):752. 29970901 10.1038/s41419-018-0715-6 PMC6030174

[48] Chen Y , Hung T , Su S , et al. MTA2 as a potential biomarker and its involvement in metastatic progression of human renal cancer by miR‐133b targeting MMP‐9. Cancers (Basel). 2019;11(12):1851. 31771219 10.3390/cancers11121851 PMC6966675

[49] Huang S , Wa Q , Pan J , et al. Transcriptional downregulation of miR‐133b by REST promotes prostate cancer metastasis to bone via activating TGF‐beta signaling. Cell Death Dis. 2018;9(7):779. 30006541 10.1038/s41419-018-0807-3 PMC6045651

[50] Zhu J , Zeng Y , Xu C , et al. Expression profile analysis of microRNAs and downregulated miR‐486‐5p and miR‐30a‐5p in non‐small cell lung cancer. Oncol Rep. 2015;34(4):1779‐1786. 26238736 10.3892/or.2015.4141

[51] Dai A , Sun H , Fang T , et al. MicroRNA‐133b stimulates ovarian estradiol synthesis by targeting Foxl2. FEBS Lett. 2013;587(15):2474‐2482. 23810756 10.1016/j.febslet.2013.06.023

[52] Benayoun BA , Georges AB , L'Hôte D , et al. Transcription factor FOXL2 protects granulosa cells from stress and delays cell cycle: role of its regulation by the SIRT1 deacetylase. Hum Mol Genet. 2011;20(9):1673‐1686. 21289058 10.1093/hmg/ddr042

[53] Del Vescovo V , Denti MA . microRNA and lung cancer. Adv Exp Med Biol. 2015;889:153‐177. 26659001 10.1007/978-3-319-23730-5_9

[54] Weidle UH , Birzele F , Nopora A . MicroRNAs as potential targets for therapeutic intervention with metastasis of non‐small cell lung cancer. Cancer Genom Proteom. 2019;16(2):99‐119. 10.21873/cgp.20116 PMC6489690 30850362

[55] Zhang H , Xie Y , Hu Z , et al. Integrative analysis of the expression of SIGLEC family members in lung adenocarcinoma via data mining. Front Oncol. 2021;11:608113. 33796453 10.3389/fonc.2021.608113 PMC8008066

[56] Jiang F , Hu Y , Liu X , Wang M , Wu C . Methylation pattern mediated by m^6^a regulator and tumor microenvironment invasion in lung adenocarcinoma. Oxid Med Cell Longev. 2022;2022:2930310. 35035657 10.1155/2022/2930310 PMC8756160

[57] Guo CR , Mao Y , Jiang F , Juan CX , Zhou GP , Li N . Computational detection of a genome instability‐derived lncRNA signature for predicting the clinical outcome of lung adenocarcinoma. Cancer Med. 2022;11(3):864‐879. 34866362 10.1002/cam4.4471 PMC8817082

[58] Tucker EJ . The genetics and biology of FOXL2. Sex Dev. 2022;16(2–3):184‐193. 34727551 10.1159/000519836

[59] Laissue P . The forkhead‐box family of transcription factors: key molecular players in colorectal cancer pathogenesis. Mol Cancer. 2019;18(1):5. 30621735 10.1186/s12943-019-0938-x PMC6325735

[60] Brunet A , Bonni A , Zigmond MJ , et al. Akt promotes cell survival by phosphorylating and inhibiting a Forkhead transcription factor. Cell. 1999;96(6):857‐868. 10102273 10.1016/s0092-8674(00)80595-4

[61] Saline M , Badertscher L , Wolter M , et al. AMPK and AKT protein kinases hierarchically phosphorylate the N‐terminus of the FOXO1 transcription factor, modulating interactions with 14‐3‐3 proteins. J Biol Chem. 2019;294(35):13106‐13116. 31308176 10.1074/jbc.RA119.008649 PMC6721940

[62] Secchi C , Benaglio P , Mulas F , Belli M , Stupack D , Shimasaki S . FOXO1 mitigates the SMAD3/FOXL2^C134W^ transcriptomic effect in a model of human adult granulosa cell tumor. J Transl Med. 2021;19(1):90. 33639972 10.1186/s12967-021-02754-0 PMC7913442

[63] Anttonen M , Pihlajoki M , Andersson N , et al. FOXL2, GATA4, and SMAD3 co‐operatively modulate gene expression, cell viability and apoptosis in ovarian granulosa cell tumor cells. PLoS ONE. 2014;9(1):e85545. 24416423 10.1371/journal.pone.0085545 PMC3887065

[64] Zeng W , Zhu JF , Liu JY , et al. miR‐133b inhibits cell proliferation, migration and invasion of esophageal squamous cell carcinoma by targeting EGFR. Biomed Pharmacother. 2019;111:476‐484. 30594787 10.1016/j.biopha.2018.12.057

[65] Sun Z , Ma Y , Chen F , Wang S , Chen B , Shi J . miR‐133b and miR‐199b knockdown attenuate TGF‐β1‐induced epithelial to mesenchymal transition and renal fibrosis by targeting SIRT1 in diabetic nephropathy. Eur J Pharmacol. 2018;837:96‐104. 30125566 10.1016/j.ejphar.2018.08.022

