# Supplementary figures and images for: Forkhead box L2 is a target of miR‐133b and plays an important role in the pathogenesis of non‐small cell lung cancer
Source: Cancer Med. 2023 Feb 27;12(8):9826–42. doi: 10.1002/cam4.5746 (PMC10166978; doi:10.1002/cam4.5746)

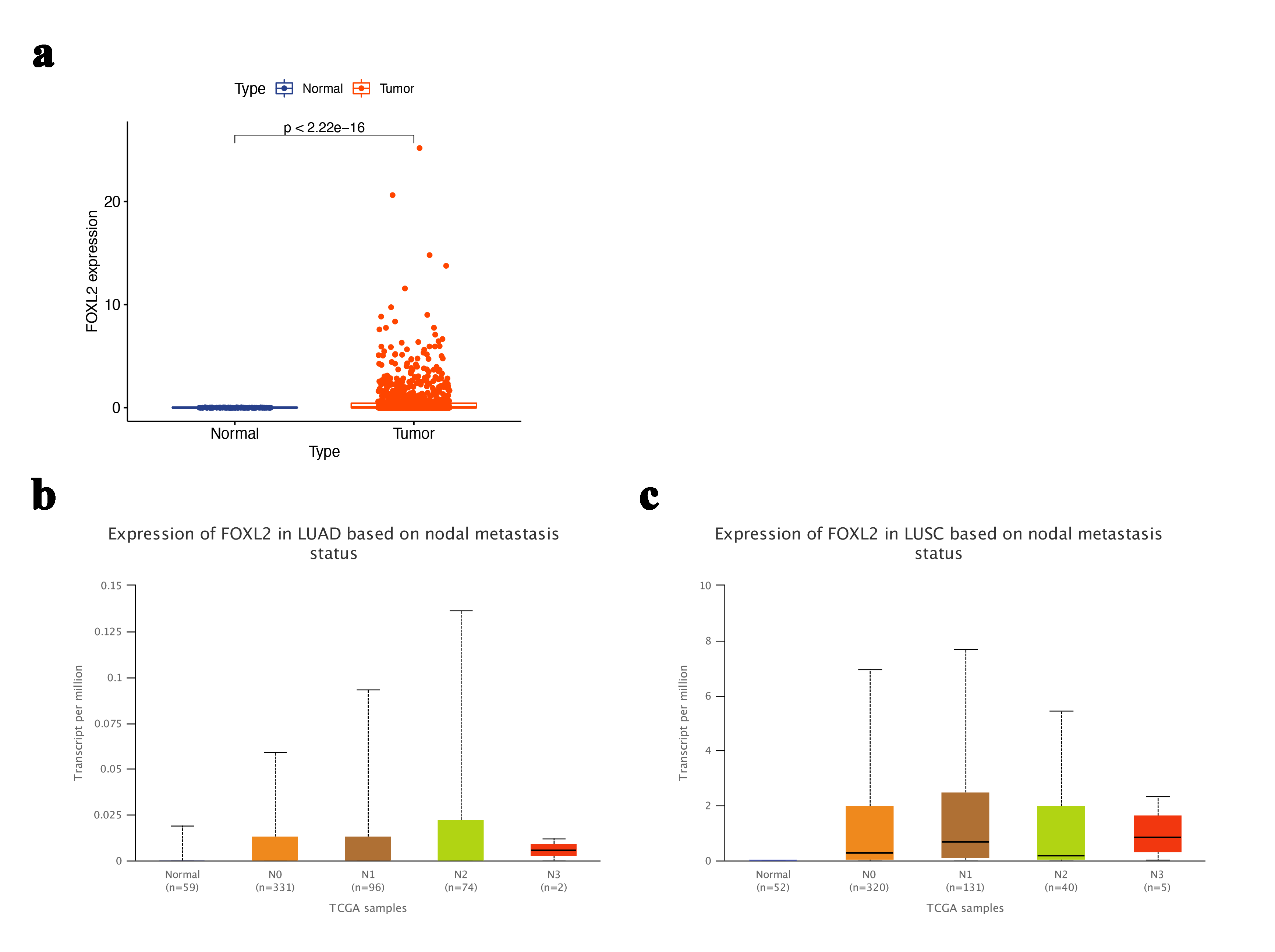

Supplement: Supplementary file 1 — Figure S1. [file CAM4-12-9826-s002.tif]

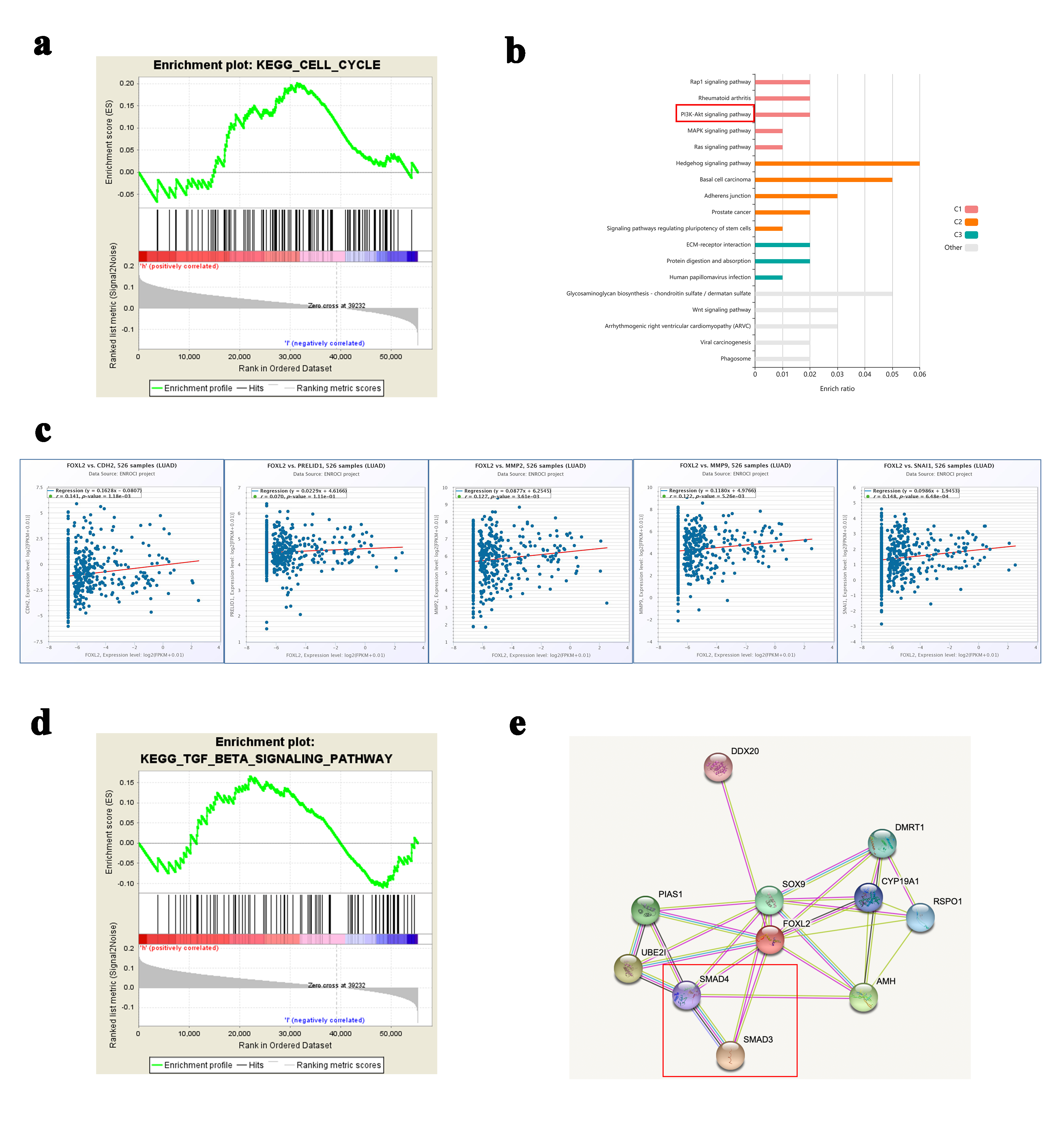

Supplement: Supplementary file 2 — Figure S2. [file CAM4-12-9826-s001.tif]

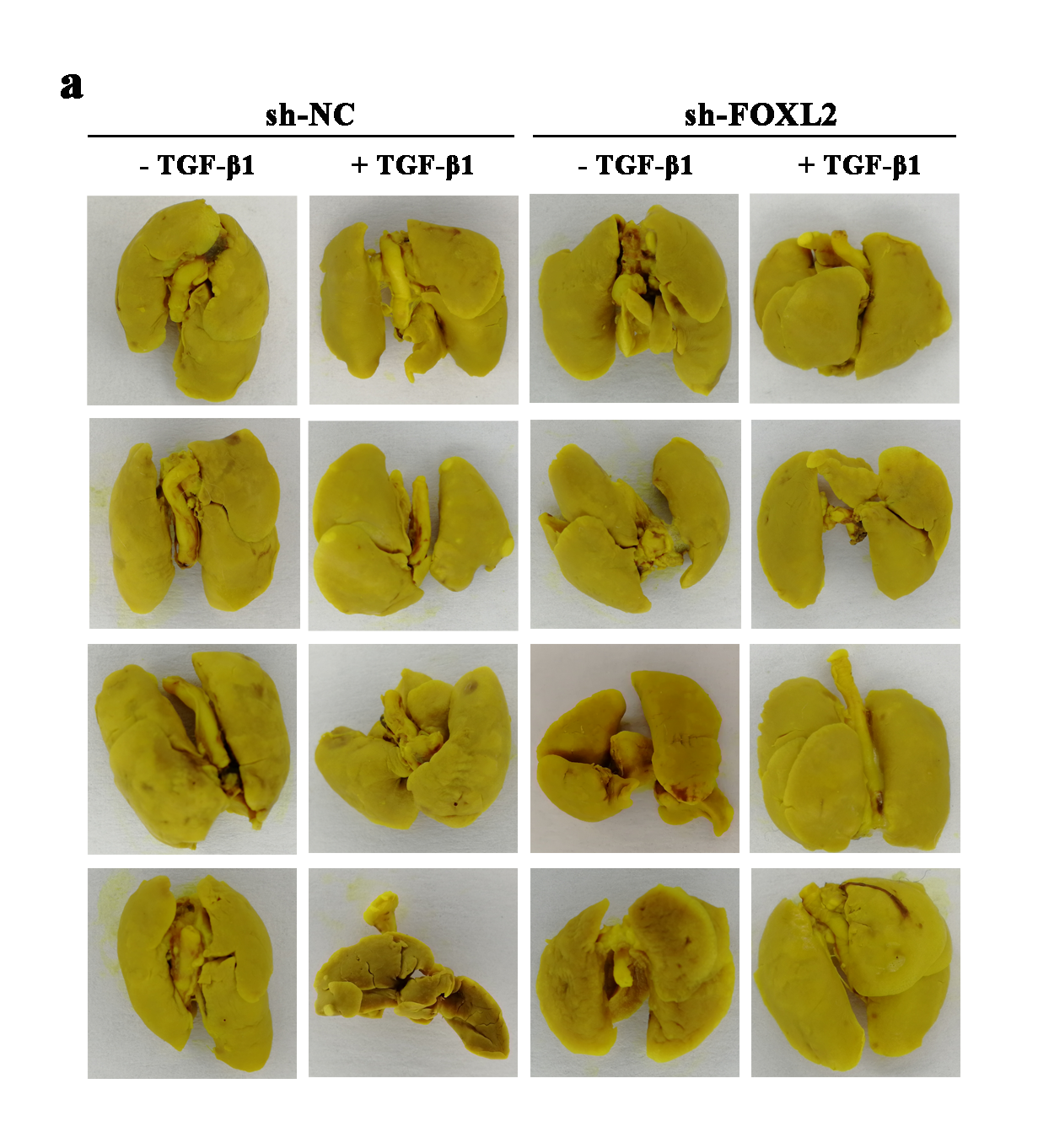

Supplement: Supplementary file 3 — Figure S3. [file CAM4-12-9826-s004.tif]

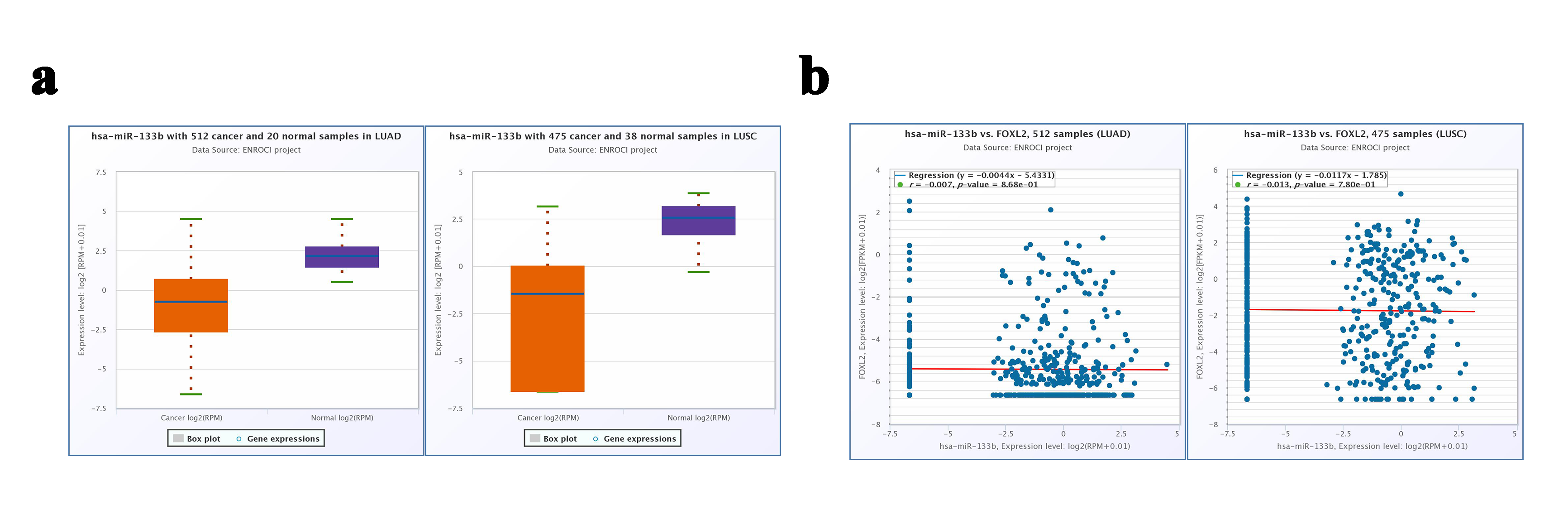

Supplement: Supplementary file 4 — Figure S4. [file CAM4-12-9826-s003.tif]
